# Experiences and Perceptions of Persons With Substance Use Disorders on Aftercare Services in KwaZulu‐Natal

**DOI:** 10.1155/oti/9598808

**Published:** 2026-07-08

**Authors:** Gugulethu Dladla, Neha Jivan, Mzwandile Mdletshe, Nokwazi Sizathina Mkhwanazi, Noluthando Pretty Ndlovu, Elizabeth Steynberg, Luther Lebogang Monareng, December Mandlenkosi Mpanza

**Affiliations:** ^1^ Department of Occupational Therapy, College of Health Sciences, University of KwaZulu-Natal, Durban, South Africa, ukzn.ac.za

**Keywords:** aftercare services, people with substance use disorders (PWSUD), substance use disorders (SUDs), substance use treatment

## Abstract

**Background:**

Aftercare is essential in sustaining recovery following substance use treatment, supporting abstinence and reintegration within the family, workplace and community. However, there is a gap between aftercare policy directives and services in South Africa, limiting access for people with substance use disorders (SUDs).

**Methods:**

This study employed a qualitative method with an exploratory research design to explore the subjective experiences and perceptions of people with SUDs using semi‐structured individual in‐depth interviews (*n* = 8). Data were analysed thematically using Braun and Clarke′s six‐phase analytic process.

**Findings:**

Four themes emerged from the study including the organisation of aftercare services, the role of aftercare services in recovery, enablers and barriers to engagement with aftercare services.

**Conclusion:**

People with SUDs regard aftercare services as valuable in strengthening relapse prevention and reintegration to families, work and communities, despite barriers to participation. We recommend that the capacity for aftercare provision be expanded, including over weekends, and that occupational therapists adopt a more central role in the delivery of these services.

## 1. Introduction

Substance use disorders (SUDs) represent a significant public health challenge globally and within South Africa, contributing to complex health, social, psychological and economic burdens for individuals, families and communities. People living with SUDs often experience disruptions in occupational functioning, relationships, mental health, physical well‐being and community participation, whereas concerned and affected others frequently face emotional strain, caregiving burden, financial stress and social marginalisation.

Substance use treatment plays a critical role in initiating recovery; however, treatment alone is insufficient to sustain long‐term recovery outcomes. Aftercare services provide structured, ongoing psychosocial, community‐based and support‐oriented interventions that assist individuals in maintaining recovery, preventing relapse, rebuilding social roles and reintegrating into family, work and community life. Effective aftercare is therefore widely recognised as a central component of recovery‐oriented systems of care.

Despite this, aftercare services in South Africa remain inconsistently structured, under‐resourced and poorly integrated within broader health and social development systems. [1] Fragmentation between treatment services, community‐based organisations and social support structures creates significant gaps in continuity of care, particularly within public sector service provision. These challenges are further compounded by inequalities between public and private healthcare systems, resulting in unequal access to sustained recovery support. [2]

Although international literature highlights the importance of aftercare in reducing relapse risk and promoting long‐term recovery, there is limited empirical research examining the practical provision, structure and lived experience of aftercare services within the South African context, particularly in KwaZulu‐Natal.

This study therefore seeks to explore how aftercare services are practically provided to people with SUDs in KwaZulu‐Natal, with the aim of informing policy development, service integration and the strengthening of recovery‐oriented care pathways.

### 1.1. Research Questions

The following questions arose once the problem was identified:

1. What are the types of aftercare services implemented for substance use in a substance use treatment facility?
2. What is the role of aftercare in community, family and work reintegration regarding relapse prevention for substance use in KZN?
3. What are the barriers to aftercare service provision for substance use in KZN?
4. What are the enablers for aftercare service provision for substance use in KZN?

## 2. Literature Review

Aftercare services refer to structured, ongoing support provided following the completion of formal substance use treatment, aimed at sustaining recovery, promoting psychosocial stability and facilitating reintegration into family, work and community life. In the South African context, aftercare typically occurs through referral pathways involving social workers, community‐based organisations, non‐governmental organisations and peer support structures, particularly within the public healthcare system. Individuals may access aftercare through outpatient services, community support programmes and recovery support groups, including 12‐step programmes.

The organisation and availability of aftercare services differ across South Africa′s dual healthcare system. Private sector services may offer more integrated and continuous care models, whereas public sector services often rely on decentralised community‐based structures. Detoxification services are understood as precursors to treatment rather than aftercare, and pharmacological maintenance programmes are not traditionally conceptualised as aftercare services within public sector systems.

### 2.1. Aftercare Services for People With SUD

The goal of SUD treatment and aftercare services is to form part of relapse prevention through tools such as reintegration into the community, families and work environments [3]. Aftercare in the context of SUDs consists of services of substance use treatment that offer help to people with SUD to adapt and function in everyday community life [3]. The Prevention and Treatment of Substance Abuse Act 70 of 2008 [4] states that aftercare helps to enable people with SUD to maintain sobriety or abstinence and is a form of ongoing support from professionals after formal treatment has ended.

These aftercare services can consist of the following:

• outpatient drug‐free treatment programmes that use strategies such as individual and group therapy
• therapeutic communities where patients with a long history of SUD will stay at a residence for 6–12 months
• short‐term residential programmes with inpatient treatment for three to 6 weeks followed by the 12‐step programme or extended outpatient treatment

### 2.2. Implementation of Aftercare Services in South Africa

In the South African context, specifically the KwaZulu‐Natal region, aftercare can be considered poor with a lack of continuity and limited successful interventions [5].

One of the principal issues in treating SUD is to maintain abstinence and recovery after treatment [6, 7]. Aftercare is fundamental for SUD for long‐term sustained sobriety, with studies showing that following inpatient treatment, 80% of recovering adults relapse [8]. Despite research proving that aftercare is crucial for improved and sustained treatment outcomes, aftercare and reintegration services remain neglected in practice and research [9]. In South Africa, little is known about the provision of aftercare services, and the emphasis in most studies in South Africa is made on treatment service provision with a lack of emphasis on aftercare [5].

Several factors lead to limited and poor service provision in aftercare. The management of long‐term substance use treatment and aftercare is often not consistently evidence‐informed, and the effectiveness of aftercare remains insufficiently evaluated. This is due to a significant lack of clear policy directions, accessibility of aftercare services, inadequate government funding in this sector and lack of awareness about policy in service providers [5]. Barriers are evident at the coordination level, control level, intelligence level, and policy level in aftercare service provision. From the perspective of service providers, they expressed a lack of internal motivation, challenges at the community level, such as stigmatisation of those with SUDs, poor community involvement in recovery and lack of resources [5]. There is still little known about the provision of aftercare services in South Africa. This study explored how aftercare services are provided on a practical level in South Africa, specifically KwaZulu‐Natal, and the nature of individual aftercare, to inform policies and services to reduce relapse rates amongst individuals in recovery.

### 2.3. Relapse Linked to Aftercare Services

Literature shows that the most common reasons identified for relapse were a lack of support from families, personal problems, environmental triggers and a lack of support from the government, specifically relating to a lack of access to aftercare services [9]. Relapse can be associated with a lack of access and/or implementation of aftercare services [10]. Another study [11] found that their results indicated that aftercare played a vital role in assisting participants to maintain treatment.

### 2.4. Role of Occupational Therapy in Aftercare

Literature shows [12] that there is a need to re‐examine the use of the disease model within aftercare services and incorporate a holistic approach which focuses on the ‘mind, body and spirit’ and makes use of a multidisciplinary team (including occupational therapy). Occupational therapy is a client‐centred health profession and works with people and communities to promote well‐being through enabling them to participate in activities of daily life and occupations that they want to, need to or are expected to do [13]. A study found that occupational therapy interventions focus on assessment, community reintegration and skills‐based therapy, which involves social and vocational skills development [14]. This study also found that an important motivator for the client to avoid relapse after community reintegration was the restoration and maintenance of roles they had lost.

A need was identified for occupational therapy practice in the transition from a 90‐day treatment programme into the community and within aftercare services [15]. This study found that occupational needs of PWSUD include social participation, meaningful leisure participation, work exploration and education, which occupational therapists are uniquely equipped to handle. It was found that participation in education and work occupations lead to greater success in the prevention of relapse and criminal activities [16].

## 3. Method

### 3.1. Research Design

The qualitative exploratory study design was used to explore subjective experiences and perceptions of persons with SUDs to meet the study objectives. This method allowed for a deeper understanding of the phenomenon of their perceptions and experiences with aftercare in substance use services through interviews with participants, where themes began to emerge [9]. Moreover, the Consolidated Criteria for Reporting Qualitative Research (COREQ) guidelines were followed.

### 3.2. Research Setting

This research was conducted with service users (PWSUD) from a substance use treatment facility, known for providing aftercare services in KwaZulu‐Natal province, a metropolitan area. The aftercare services consist of a weekly meeting on a Monday evening with outpatients and social workers in attendance. This facility therefore offers an opportunity to explore perspectives and experiences of PWSUD on their weekly aftercare services.

### 3.3. Participants

Eight male participants ranging in age from 31 to 47 participated in the study. The sample comprised people attending aftercare services for an SUD to meet the study objectives. All participants were outpatients of the substance use treatment facility and had at least one prior admission to aftercare. See Table 1 for the demographic profile of participants.

**Table 1:** The demographic profile of the participants.

Pseudonym	Age	Ethnicity	Gender	Number of treatment admissions	Marital status	Residence	Employment status	Type of employment	Highest level of education	Duration of recovery	Previous substances	Amount of previous relapses
Mike	47	Black	Male	1	Divorced	KwaZulu‐Natal	Employed	Process controller	Matric	2 years	Alcohol	1
Mzwandile	37	Black	Male	1	Single	KwaZulu‐Natal	Employed	Information technology (IT)	Tertiary	10 months	Alcohol	1
Faruk	32	Indian	Male	2	Married	KwaZulu‐Natal	Self‐employed	Manufacturing	Matric	3 months	Alcohol, cocaine and rohypnol	2
Henry	40	Black	Male	1	Married	KwaZulu‐Natal	Employed	Finance officer	Tertiary	6 years	Alcohol and Ecstasy	1
David	31	Black	Male	3	Single	KwaZulu‐Natal	Employed	Production team leader	Tertiary	2 years	Alcohol, cannabis and cocaine	3
Zayne	38	Indian	Male	2	Single	KwaZulu‐Natal	Unemployed	Not applicable	Tertiary	1 month	Cocaine	5
Sani	31	Black	Male	1	Single	KwaZulu‐Natal	Employed	Process technician	Tertiary	2 years	Alcohol, cocaine, ecstasy and methamphetamine	3
Vukile	32	Black	Male	1	Single	KwaZulu‐Natal	Employed	Security	Matric	1 month	Alcohol and cannabis	0

To select participants, purposive and convenience sampling were used. Purposive sampling was used to recruit participants who were directly involved in the provision and/or utilisation of aftercare services for people with SUDs in KwaZulu‐Natal.

Convenience sampling is an approach used by qualitative researchers to select participants who are easy to reach [17].

### 3.4. Recruitment Process

After receiving ethical clearance and gatekeeper permission, the researchers emailed the substance use treatment facility to ask for permission for service users (PWSUD) to join their study and to determine a suitable period to conduct research with participants. The researchers approached the substance use treatment facility in the city to interview participants. The participants were recruited through social workers informing them about the study and the inclusion criteria during aftercare sessions. Written consent was obtained from each prospective participant after being informed about the study.

### 3.5. Data Collection

The data collection process, between 11 August and 30 September 2023, followed a qualitative approach where perspectives and experiences of PWSUD were explored through the use of semi‐structured individual interviews. An interview guide that had six broad questions was developed by the researchers and piloted within the field of occupational therapy. Revisions made after the pilot were the addition of probing questions to meet objectives and assist in directing questioning. Researchers conducted and audio‐recorded a total of one semi‐structured individual interview per participant in person or via Zoom, of a duration of 20–45 min. To ensure confidentiality throughout the research process, no identifiable information was collected. For example, video functions were disabled, and pseudonyms were used during Zoom meetings. Through these interviews, the perceptions and experiences of PWSUD on the implementation of aftercare services were obtained. The data collected were stored on a shared Google Drive, with access restricted to the researchers.

### 3.6. Data Analysis

In the data analysis process, audio recordings were transcribed using AI technology. The transcripts were then edited and finalised by listening to the audio recording simultaneously. The data were then analysed using the thematic analysis method. This method of analysing data was used by the researcher to analyse data to find recurrent themes, topics, notions and patterns of meaning in a collection of texts such as interviews and transcripts [18]. The data collected were analysed using the six‐phase analytic process by Braun and Clarke [19].

Audio recordings were transcribed and reread multiple times for the researchers to familiarise themselves with the data and identify patterns. The next phase included generating codes for the data through the hybrid approach. There were a total of 18 codes identified with 11 being inductive and seven being deductive. Themes were then constructed from the codes and influenced by the research question. This involved identifying similarities across different codes and combining them to form four main themes. The data and themes that were developed were then reviewed to ensure that they captured the data that were coded and that it is relevant to the research question with no repetition of information. The themes were named and defined by summarising the core meaning and content of the themes that were formulated. From a total of eight in‐depth interviews conducted, data saturation was achieved by the eighth participant, at which point no new meaningful insights emerged, and a sufficient understanding of participants′ perceptions and experiences had been reached. A final analysis of data codes and themes that were developed was put together to produce a final report of the findings.

### 3.7. Trustworthiness

Trustworthiness was ensured through credibility, confirmability, dependability and transferability. This was done through providing verbatim quotes from participants, using data triangulation where data were being reviewed by multiple researchers, regular debriefings with peers and supervisors and an audit trail was utilised to establish confirmability. Additionally, the study presents detailed descriptions of the research context and methods. A description of methods and findings was provided to establish the transferability of this study.

### 3.8. Ethical Clearance and Considerations

Ethical approval was obtained from an accredited Research Ethics Committee, the Biomedical Research Ethics Committee of the University of KwaZulu‐Natal, with protocol reference number: BREC/00005799/2023. Gatekeeper permission was received from the aftercare team at the substance use treatment facility.

The ethical considerations of autonomy, informed consent and confidentiality were practised throughout this study. Each participant chose to participate out of their own free will; they were informed of all relevant information and potential risks; written consent was obtained from all participants; and all identifying and confidential information was kept private through the use of pseudonyms or excluding information.

## 4. Findings

The findings are presented in accordance with emergent themes relating to the predetermined objectives. As depicted in Table 2 below, the first theme describes the types of aftercare services, followed by the role of aftercare services and an exploration of the enablers of aftercare services, concluding with the theme of barriers that hinder aftercare services.

**Table 2:** Themes and subthemes.

Themes	Subthemes
Organisation of aftercare services	Structure of aftercare services
	Routine drug testing
The role of aftercare services in recovery	The role of aftercare in relapse prevention
	The role of aftercare in family, work and community reintegration
Enablers to aftercare services	Promotion of aftercare services
	The use of external services
Barriers to aftercare services	Accessibility to aftercare services
	The relapse of fellow service users

### 4.1. Theme 1: Organisation of Aftercare Services

#### 4.1.1. Subtheme: Structure of Aftercare Services

Participants shared that they attend aftercare sessions held once a week on a Monday, having prepared to discuss experiences in their sobriety journey. They had positive experiences due to sessions being well‐organised, creating familiarity and comfort as they engaged in sessions that consisted of discussions sharing their experiences, strengths and challenges with social workers and fellow service users. These discussions served as a platform for providing coping skills to address various situations encountered during the week. A participant also mentioned that having sessions on a Monday proved beneficial to address recently experienced challenges and emotions that are most prominent during weekends.

*So, we attend on Mondays, we share how the week was from Tuesday until Sunday. How are we feeling, how are we coping with life in general? Are there any struggles?—David (31)*

*So, knowing that I have to come here has brought some sort of structure to my life that there are certain things that I cannot do because I have to be here.—Mzwandile (37)*

The aftercare sessions consist of a group, either in person or online. These sessions offer a range of services, such as counselling, support groups and life skills training. There is also a 24‐h hotline where participants can seek help if required. This social support and mentorship encourage participants to stay motivated as they navigate their goals.

*At any given time, you come here with your shoulders heavy and you know, you′ve got people whereby you can talk to. They′ll listen, they′ll give suggestions, they′ll give you genuine opinions.—Mike (47)*

#### 4.1.2. Subtheme: Routine Drug Testing

Participants mentioned the incorporation of random drug tests as a valuable component in maintaining sobriety. One participant discussed the process of random drug testing at the end of aftercare sessions, where social workers would selectively choose participants for urine tests.

*One of the benefits also that we get from the aftercare sessions is there are routine drug tests that are done randomly. You′re not told, but then at the end of the aftercare session, the social worker will just randomly pick whoever they feel or would see it′s been a while since they′ve done a drug test and then we′ll go and do that urine test at the end of the session.—Sani (31)*

Participants expressed that the unpredictability of these tests was a crucial factor in helping them to stay in recovery and avoid substance use. This practice serves to ensure that individuals are not attempting to bypass the system, therefore re‐enforcing that participants need to take accountability while on their journey.

*I think that drug test does help a lot because you don′t know when you′re going to be tested.—Zayne (38)*

### 4.2. Theme 2: The Role of Aftercare Services in Recovery

#### 4.2.1. Subtheme: The Role of Aftercare in Relapse Prevention

Aftercare programmes provide ongoing support and guidance to individuals as they transition back into society. Participants expressed the transformational impact of aftercare on their lives. It is often described as the central core of their recovery, helping them sustain their sobriety and navigate everyday challenges outside of rehab.

*Aftercare, I think is aftercare, how can I put it? Is the central core of my recovery. It′s where my journey of recovery started.—David (31)*

*If you want to go forth and you want to live in sobriety and you want to do well in recovery, you got to make this your minimum requirement to say, look, I′m going to attend after care, but you′ve got to build on it further.—Faruk (32)*

Participants viewed their relationship with one another as one of the supporting structures in maintaining their sobriety or preventing relapse. Additionally, participants expressed that these relationships and support helped them develop coping mechanisms and build resilience, ultimately enabling them to navigate challenges and triggers outside of the treatment environment. This was done through the sharing of experiences, providing encouragement, holding themselves accountable, and social connection.

*…and there′s power in listening to someone who′s going through the same thing more so than the professional. Yes, professionals definitely assist us when we get stuck, but there′s definitely power from listening to someone who shares the same sentiments as we do.—Henry (40)*

*So I have a solution and I have a problem without me actually having been into that problem. And already when it comes, I have already listened to another person′s perspective on the solution and I can use my own experiences as well to sort of then work through the solution of that particular issue.—Henry (40)*

The participants also emphasised the importance of social workers′ consistent presence and availability. Participants expressed that having support from social workers who were constant in their support played a huge role in their recovery journey in aftercare as it helped them feel understood and motivated to continue their recovery efforts. Additionally, ongoing support provided by these social workers helped build a sense of trust and rapport, creating a safe space for participants to openly discuss their challenges and seek guidance.

*The social workers in the aftercare facility, they believed in me. I could see, that they really wanted me to succeed in this. You have someone who believes in you, who′s always reminding you the amount of effort they put in my recovery, it kept me wanting to keep coming back.—Sani (31)*

#### 4.2.2. Subtheme: The Role of Aftercare in Family, Work and Community Reintegration

The participants have all mentioned that involvement of their families within aftercare services has been beneficial in restoring relationships and trust that was lost during their time of substance use. The aftercare services have regular sessions where family members are asked to attend alongside the service users, where counselling and group therapy are done to aid with reintegration into their families following the substance use treatment process.

*…you brought your family and to be part of the part of this programmes. And they learn a lot, to recover themselves because when we′re doing all this stuff, they get affected, they get affected a lot. And the aftercare have a programme to heal your family too.—Vukile (32)*

*In my mind, my wife was fully aware of the situation. She′s been through the programme, she′s been family groups and stuff, so she was aware she′d support me.—Faruk (32)*

*So, with those family meetings, they discussed coping skills and they got the families there and they discussed to them that the problem we have and how to deal with us and what they going through and things like that.—Zayne (38)*

Some participants have mentioned that support from their family following their decision to start the recovery process and attend aftercare has also been a source of great encouragement. The aftercare services also focus on the importance of having to rebuild trust within relationships with family members and loved ones.

*So, they were so supportive. At home, my parents would lend me their car. The trust was back.—Sani (31)*

*But they teach you that when you go back into society and you go back to your communities or to your workplace or to your homes, there′s a way that you ought to be you shouldn′t be expecting everyone to be like, wow, you′ve changed and stuff. But you′ve got to work through and I wouldn′t say win back people′s trust, but I mean, that′s part of it.—Faruk (32)*

Participants have also spoken about how social workers at aftercare services involve their previous employers and managers by updating them regularly on their recovery and process to ensure that the participants can continue to work and not let them lose their position within the company. One participant in particular spoke on communication between the social workers and his manager and how his employer is now allowing him to come to aftercare services on a Monday between work hours.

*They′ll ask even maybe your immediate manager or what, they′ll have communication with him or her, to give them the feedback, progress on your recovery while you′re still here. So, by the time you go back to work, they have a little bit of understanding of what is happening with you. So, yeah, definitely, they did play a role in that, going back to work…—Mike (47)*

Some participants expressed fear that they experienced with reintegration into the community. They mentioned being frightened of the stigma that comes with being a recovering person with an SUD; how will they be perceived by people they used to share this lifestyle with, how will they regain trust from loved ones and how will they reintegrate into the community having lost many friends.

*So that has been my fear of how they are going to treat me post my drinking? How are they going to perceive me if I′m no longer part of the social life that we used to be engaged in before?—David (31)*

Participants shared that aftercare services assisted them in regaining their confidence in terms of community service. Participants shared that being of service during their sobriety within the community is how they reintegrated themselves into society. This was done by building trust with people that they need to interact with for effective reintegration.

*So HNI is hospitals and institutions where if you′re above six months clean, you can join that committee and you go and be of service, where we go and visit rehab, we give talks, we go to jails, we go to hospitals. And we share that message of hope. The same message of hope that I received when I was in rehab.—Sani (31)*

*But the more you keep coming back to the aftercare sessions, it taught me to regain this confidence and also be able to talk out loudly, talk in front of people, to not be afraid to talk. So, after care taught me that, taught me to build my confidence, taught me to talk in front of people. In terms of reintegration into society.—Sani (31)*

### 4.3. Theme 3: Enablers to Aftercare Services

#### 4.3.1. Subtheme: Promotion of Aftercare Services

The biggest source of referral for these aftercare services is in the form of experienced service users and social workers recommending aftercare while they are still within the 21‐day substance use treatment process. This encourages the service users to attend aftercare services as it is recommended through trusted sources as both experienced service users and social workers understand their difficulties and situations and are therefore able to make realistic recommendations.

*It was through the social workers. It′s something that they speak about. It′s something that they promote, and they place a lot of value in it because they are the ones that have dealt with each individual′s case, and they understand what needs to go through and go on in the person′s life.—Faruk (32)*

*So, I had a guy who I′m still close friends with who was an ex patient and who is also in a programme…He suggested as well as the social workers, my individual social worker, who I found at the rehab suggested that I take part in the aftercare session….—Sani (31)*

The participants mentioned that they were encouraged to see the recovery of experienced service users and would join aftercare services in the hope of also having the same road to recovery and sobriety. It gave them hope to see that people coming from similar difficulties and situations were able to find a way to maintain their sobriety through the use of aftercare services.

*And also, the hunger, when I saw the guys who came before me in the aftercare session, when I saw how happy they are, when I saw the things that they were achieving, when I saw how handsome they looked, when I saw how they were prospering in life, that motivated me to wanting to stay.—Sani (31)*

#### 4.3.2. Subtheme: The Use of Alcoholics Anonymous and Narcotics Anonymous Services

External support from aftercare services such as Alcoholics Anonymous (AA) and Narcotics Anonymous (NA) is also a resource that all participants have mentioned that they make use of. It was mentioned that the time between weekly aftercare meetings has made it necessary for participants to seek additional support from sources such as AA and NA, as a week can pose many challenges where they might need assistance before the next aftercare meeting.

*I′ve been engaged in the NA Fellowship, and they speak about that a lot in aftercare. They promote it a lot in aftercare. Like I said, you′re coming on a Monday to aftercare, but there′s six other days in the week, and anything could happen along the way. It′s been a long time.—Faruk (32)*

### 4.4. Theme 4: Barriers to Aftercare Services

#### 4.4.1. Subtheme: Accessibility to Aftercare Services

Accessibility to attend these aftercare services has been mentioned to be hindered by obstacles such as transport, working hours, studies and time management. Some participants have mentioned that they have observed that some service users live too far away from the aftercare services to attend meetings on a regular basis. Another participant also mentioned that their working hours interfere with the time of aftercare meetings and that they cannot always arrange for accommodations to leave work for that time.

*There are no excessive struggles that I know of, except that we work six to six. Sometimes time management, I think, based on the demand at work, maybe sometimes I′ll knock off late at work, so I wouldn′t attend the physical meeting.—David (31)*

The aftercare services have, however, made a concentrated effort to overcome these barriers by providing additional online aftercare meetings via Zoom on a Monday evening. Some service users also volunteer to pick up other members to ensure that all who wish to attend are given the opportunity.

*I mean, there′s guys who volunteer to pick up other guys to come through for after care, and for the ones that obviously can′t make it through, there is this online Zoom meeting for the guys who can′t.—Faruk (32)*

Although the facility has made it possible for service users to access meetings via online Zoom meetings, challenges such as load shedding in South Africa that disrupt the network limit service users′ ability to access and engage within the meeting. Online meetings also posed a challenge during the COVID‐19 pandemic, with two participants mentioning that they lost interest in attending aftercare meetings and one of these participants mentioning how he experienced a relapse due to this factor.

*But as soon as things got a bit rocky and there was like your lockdown in place and stuff, and there were meetings on the Zoom, I began to lose interest. And I think that led to the beginning of a relapse the second time.—Faruk (32)*

*…it was still COVID times, so they did not have any face-to-face meetings, so we only relied on Zoom meetings, online meetings. I don′t like online meetings. I want face to face, I want interaction. So, there would be challenges of network and poor connection….—Sani (31)*

#### 4.4.2. Subtheme: The Relapse of Fellow Service Users

A theme that arose from multiple interviews with participants is the phenomenon of service users attending aftercare sessions for a few meetings and then stopping attending. One participant mentioned that the reason for this is often because of relapses, whereas two others were of the belief that they stop attending once they no longer recognise the faces that attended substance use treatment with them.

*What I′ve noticed is people are very eager for the first four Mondays after being discharged from rehab. They′re constantly here, and then people just vanish. People relapse literally within a month.—Mzwandile (37)*

*…most of the guys who I′ve met the first time and the second time being here, they′d come for aftercare, like, once or twice just to see their friends, the guys they were in patients with, and then slowly they fall away.—Faruk (32)*

*So, they′ll come the first Monday, the second Monday, the third Monday, they gone. Because if they come back, no one knows them. They only want to stand there and talk to the people they left here.—Mike (47)*

The participants mentioned that they are discouraged by seeing past users′ discontinuation from aftercare services due to relapse, which triggers a range of emotions. They feel sympathetic, anxious and terrified about their own relapse risk.

*…sometimes it′s not nice in an aftercare session when you listen to someone else share about how difficult they are finding it to stay clean. Like when someone comes and they share that they relapsed. It′s heartbreaking because I know the pain.—Sani (31)*

Some service users view relapse as a setback but use it as a learning opportunity to recommit to their recovery journey; however, other service users become demotivated and feel discouraged. The service users who have discontinued due to relapse experience feelings of disappointment, guilt, shame, and frustration. This then negatively affects their volition to return to the program.

## 5. Discussion

### 5.1. Types of Aftercare Services

The study confirmed that structured weekly programmes promoted attendance, adherence and active participation by attendees. Ekendahl [20] explores how social workers perceive aftercare in the context of compulsory treatment, describing it as a permanent process aimed at improving the living conditions of clients. Mowbray [21] identifies various treatment settings for older adults with substance use problems, including primary care settings, combined individual and group‐based settings, individual‐based treatment settings and multiple treatment/multisite settings. Moreover, it was also found that structured aftercare programmes were associated with increased attendance and reduced substance use at follow‐up [22, 23]. An increase in aftercare sessions carried out throughout the week at this specific facility and the introduction of aftercare services in more substance use treatment facilities in KwaZulu‐Natal would therefore prove beneficial to the outcomes of people who use substances in recovery.

### 5.2. The Role of Aftercare in Relapse Prevention

The study concurred with literature [24] in that service users evaluated a relapse prevention group positively and felt that it helped them recover post‐treatment. Interpersonal process aftercare groups have resulted in comparable improvement in people who use substances′ outcomes and aftercare attendance. This study supports that aftercare is core to recovery and provides a safe space to express oneself.

The study revealed that the foundation of family reintegration is regaining lost trust due to substance use. This can be reiterated by a study [10] which demonstrated that partners of nyaope users emphasised the need for people who use substances to regain the trust of the family and community by changing their lifestyle choices.

The study also showed that service users understood that they had to make new lifestyle choices in terms of their roles and responsibilities in the family and community for effective reintegration. This includes new lifestyle choices about people they associate with in the community, the services they render to the community, commitment and consistency. Participants have seen the importance and need for being of service to the community, thus engaging in responsibilities such as being members of AA and instilling a sense of hope in other recovering people with SUDs. Literature [10] demonstrated that it was crucial for people who use substances to assume new roles and responsibilities when reintegrating into the community.

### 5.3. Enablers to Aftercare Services

Studies have shown that having supportive relationships is a strong protective factor against mental illnesses and helps to increase mental well‐being [25]. The study shows that internal support of professionals and peers within aftercare services is required. Peer support in recovery groups has been linked to better substance use outcomes and overall well‐being [26]. This finding supports the study′s findings that support from aftercare services is a crucial component of recovery and maintaining sobriety. The value of peer support groups, such as 12‐step programmes like AA and NA, in assisting people in maintaining their sobriety has been noted in several studies, as they openly share their experiences and give advice to one another, fostering a strong sense of emotional support. It was found that regular attendance at AA meetings was linked to longer periods of sobriety [26]. Another study [27] highlighted that emotional support from family and friends can reduce stress and emotional distress, making it less likely for people to turn to substances for relief, as stress is frequently a trigger for relapse. People are less prone to return to using drugs when their stress levels are lower [26]. The social workers who are involved in aftercare have made sure to effectively fulfil this role by educating people about substance use, its triggers, and relapse prevention techniques. Research has shown that informed people are better able to make decisions that support their recovery.

### 5.4. Barriers to Aftercare Compliance

The unavailability of transport reduces access for service users who are unable to organise transport outside the city around the time of the meeting, ultimately affecting their compliance with aftercare services. This is accompanied by the inability to virtually meet due to connectivity disruptions caused by load shedding, which further acts as a barrier to accessing aftercare services experienced by service users.

The discontinuation of former service users due to relapse highlights the need for continuous education and support within the programme. It reinforces the message that recovery is not always a linear process [28]. This also serves as a learning opportunity for current service users. They gain insights into the challenges of recovery and the importance of ongoing commitment. It may lead to the implementation of additional resources and strategies to assist the service users in avoiding relapse and address it more effectively when it occurs.

## 6. Limitations of the Study

This study had two main limitations: the lack of female participants and a low response rate. All participants were male, which limits the understanding of female experiences of SUD in aftercare services, despite evidence that women often present with higher vulnerability profiles and are less likely to access treatment, even though prevalence rates are higher in men. Future studies should actively recruit female participants through increased awareness and direct engagement at facilities. Additionally, scheduling difficulties between participants′ work commitments and students′ availability contributed to a low response rate, increasing the risk of non‐response bias and limiting the representativeness of the findings. Resource and service availability differences between public and private healthcare systems may further limit transferability.

Additionally, although the consent form stated that all interviews would be audio‐recorded for data analysis, it did not explicitly mention the use of AI‐assisted transcription. Moreover, although the data were stored securely and manually verified by the research team, we acknowledge this as a limitation and recommend that future consent forms explicitly include the use of AI transcription tools. Despite these limitations, the study provides valuable insight into the lived realities of aftercare provision and recovery support in KwaZulu‐Natal.

## 7. Conclusion

In conclusion, this study explored the experiences and perceptions of persons with SUDs of aftercare services in a substance use treatment facility based in KwaZulu‐Natal, South Africa. The type of aftercare that is implemented within a facility in KwaZulu‐Natal was found to consist of weekly group meetings with service users and social workers in person and via online meetings. The experiences of participants were found to be positive regarding the assistance of aftercare services in preventing relapse and integrating them back into their families, work and communities. It was also found that the enablers of the social workers and fellow service users outweighed barriers of accessibility and witnessing the relapse of fellow service users.

## 8. Recommendations

### 8.1. Increase the Awareness and Implementation of Aftercare Services Across KZN

The social workers involved should spread awareness about aftercare services with the aim of increasing the implementation of services across KZN. Larger multidisciplinary teams should be involved in aftercare services at more substance use treatment facilities, thereby also increasing the number of individuals who seek and receive these services. This would in turn reach a broader population and address geographical barriers to increase accessibility to aftercare services.

### 8.2. Implications for Occupational Therapy Practice

Occupational therapists should be incorporated into aftercare services as they focus on helping individuals regain skills and routines necessary for daily life. Their theory and practice can significantly contribute to relapse prevention and successful reintegration into society by addressing vocational, educational, and social aspects of recovery as well as promoting healthy routines and stress management. Their inclusion in aftercare services will provide a holistic and client‐centred approach, improving long‐term outcomes for individuals in recovery.

### 8.3. Implications for Future Research

Future research should prioritise the structural organisation and coordination of aftercare services within public sector systems, with particular focus on continuity of care, intersectoral collaboration and referral pathways between treatment facilities and community‐based services. Further investigation into community‐level recovery support models, family involvement in recovery processes and the role of social capital in sustained recovery is recommended. Research exploring policy implementation gaps and systemic barriers to accessible aftercare provision is also essential for strengthening recovery‐oriented service systems.

## Author Contributions

Luther Lebogang Monareng and December Mandlenkosi Mpanza supervised the research group and assisted with guidance and critical reviews throughout the writing process of the manuscript.

## Funding

No funding was received for this manuscript.

## Conflicts of Interest

The authors declare no conflicts of interest.

## Data Availability

The data that support the findings of this study are available from the corresponding author upon reasonable request.

## References

[1] Substance Abuse in SA: The Critical Need for More Usable Research, 2022, https://www.bapsa.net/single-post/substance-abuse-in-sa-the-critical-need-for-more-usable-research.

[2] Myers B. J. , Louw J. , and Pasche S. C. , Inequitable Access to Substance Abuse Treatment Services in Cape Town, South Africa, Substance Abuse Treatment, Prevention, and Policy. (2010) 5, 10.1186/1747-597X-5-28, 21073759. PMC2992042 21073759

[3] Mittal S. , Aftercare Services for Drug Dependent Persons, 2002, United Nations Office on Drugs and Crimes (UNODC).

[4] Government Gazette , Prevention of and Treatment for Substance Abuse Act (70 of 2008), 2009, Pretoria.

[5] Mpanza D. M. , Govender P. , and Voce A. , Perspectives Of Service Providers On Aftercare Service Provision For Persons With Substance Use Disorders At A Rural District In South Africa, Substance Abuse Treatment. (2022) 17, no. 1, 10.1186/s13011-022-00471-5, 35962363. PMC9373456 35962363

[6] Lash S. J. , Burden J. L. , Monteleone B. R. , and Lehmann L. P. , Social Reinforcement of Substance Abuse Treatment Aftercare Participation: Impact on Outcome, Addictive Behaviors. (2004) 29, no. 2, 337–342, 10.1016/j.addbeh.2003.08.008, 14732421. 14732421

[7] Orbon M. , Mercado J. , and Balila J. , Effects of Forgiveness Therapy on Recovery Among Residents of Drug Rehabilitation Centers, Procedia—Social and Behavioral Sciences. (2015) 165, 12–20, 10.1016/j.sbspro.2014.12.599.

[8] Jacobs D. , The Availability and Accessibility of Aftercare Services for Recovering Adult Addicts in the Western Cape, 2021, pHD diss,. Stellenbosch University.

[9] Mahlangu S. and Geyer S. , The Aftercare Needs Of Nyaope Users: Implications For Aftercare And Reintegration Services, Social Work Journals. (2018) 54, no. 3, 327–345, 10.15270/54-3-652.

[10] McKay J. R. , Effectiveness Of Continuing Care Interventions For Substance Abusers, Evaluation Review. (2001) 25, no. 2, 211–232, 10.1177/0193841X0102500205. 11317717

[11] Elias S. and Padmanabhanunni A. , Rehabilitated Substance Abusers’ Experience of Aftercare Following Completion of Inpatient Treatment, 2016, University of the Western Cape.

[12] Jeewa A. and Kasiram M. , Treatment for Substance Abuse in the 21st Century: A South African Perspective, South African Family Practice. (2008) 50, no. 6, 44–44d, 10.1080/20786204.2008.10873782.

[13] World Federation of Occupational Therapists [Internet], 2017, https://wfot.org/about/about-occupational-therapy.

[14] Bell T. , Wegner L. , Blake L. , Jupp L. , Nyabenda F. , and Turner T. , Clients′ Perceptions of an Occupational Therapy Intervention at a Substance Use Rehabilitation Centre in the Western Cape, South African Journal of Occupational Therapy. (2015) 45, no. 2, 10–14, 10.17159/2310-3833/2015/v45n2a3.

[15] Cruz T. , Identifying Occupational Therapy Role for Individuals in Substance Abuse and Addiction Recovery Programmes, 2019, pHD diss., |University of St Augustine for Health Sciences, 10.46409/sr.FORS9739.

[16] Manuel J. I. , Yuan Y. , Herman D. B. , Svikis D. S. , Nichols O. , Palmer E. , and Deren S. , Barriers and Facilitators to Successful Transition From Long-Term Residential Substance Abuse Treatment, Journal of Substance Abuse Treatment. (2017) 74, 16–22, 10.1016/j.jsat.2016.12.001, 28132695. 28132695 PMC5310811

[17] Etikan I. , Comparison of Convenience Sampling and Purposive Sampling, American Journal of Theoretical and Applied Statistics. (2016) 5, no. 1, 1–4, 10.11648/j.ajtas.20160501.11.

[18] Caulfield J. , How to Do Thematic Analysis | Step-by-Step Guide & Examples [Internet], 2019, https://www.scribbr.com/methodology/thematic-analysis/.

[19] Braun V. and Clarke V. , Conceptual and Design Thinking for Thematic Analysis, Qualitative Psychology. (2022) 9, no. 1, 3–26, 10.1037/qup0000196.

[20] Ekendahl M. , Aftercare and Compulsory Substance Abuse Treatment: A Venture With Potential?, Contemporary Drug Problems. (2007) 34, no. 1, 137–161, 10.1177/009145090703400107.

[21] Mowbray O. and Quinn A. E. , A Scoping Review of Treatments for Older Adults With Substance Use Problems, Research on Social Work Practice. (2016) 26, no. 1, 74–87, 10.1177/1049731515579075.

[22] Ito J. , Donovan D. , and Hall J. , Relapse Prevention in Alcohol Aftercare: Effects on Drinking Outcome, Change Process, and Aftercare Attendance, Addiction. (1988) 83, no. 2, 171–181, 10.1111/j.1360-0443.1988.tb03978.x. 2830931

[23] Sannibale C. , Hurkett P. , Van Den Bossche E. , O′Connor D. , Zador D. , Capus C. , Gregory K. , and Mckenzie M. , Aftercare Attendance and Post-Treatment Functioning of Severely Substance Dependent Residential Treatment Clients, Drug and Alcohol Review. (2003) 22, no. 2, 181–190, 10.1080/09595230100100624. 12850905

[24] Anand I. , Smith A. , Charge K. J. , and Kouimtsidis C. , Are We Doing Enough to Enhance Attendance to Aftercare? Service Users′ Evaluation of a Group Relapse Prevention Programme for Alcohol Dependence, Drugs and Alcohol Today. (2015) 15, no. 1, 9–11, 10.1108/DAT-11-2014-0038.

[25] Building a Supportive Network, 2023, https://www.healthhub.sg/live-healthy/buildingasupportivenetwork#:%7E:text=It%2520is%2520important%2520to%2520surround.

[26] Turpin A. and Shier M. L. , Peer Support and Substance Use Disorder Treatment: Benefits and Barriers for Intra-Personal Development in Longer-Term Treatment Programs, Journal of Groups in Addiction & Recovery. (2017) 12, no. 2-3, 117–134, 10.1080/1556035X.2016.1258683.

[27] Daley D. C. , Family and Social Aspects of Substance Use Disorders and Treatment, Journal of Food and Drug Analysis. (2013) 21, no. 4, S73–S76, 10.1016/j.jfda.2013.09.038, 25214748. 25214748 PMC4158844

[28] White W. L. , Relapse Is NOT a Part of Recovery, 2010, https://deriu82xba14l.cloudfront.net/file/353/2010-Relapse-is-NOT-Part-of-Recovery.pdf.

